# Implementation of a Hardware-Assisted Bluetooth-Based COVID-19 Tracking Device in a High School: Mixed Methods Study

**DOI:** 10.2196/39765

**Published:** 2023-04-07

**Authors:** Dan Li, Tyler Shelby, Marie Brault, Rajit Manohar, Sten Vermund, Ashley Hagaman, Laura Forastiere, Tyler Caruthers, Emilie Egger, Yizhou Wang, Nathan Manohar, Peter Manohar, J Lucian Davis, Xin Zhou

**Affiliations:** 1 Yale School of Public Health Yale University New Haven, CT United States; 2 Yale School of Engineering and Applied Science New Haven, CT United States; 3 IBM T.J. Watson Research Center Yorktown Heights, NY United States; 4 Carnegie Mellon University Pittsburgh, NY United States

**Keywords:** contact tracing, COVID-19, digital contact tracing, Bluetooth device, school health, secondary school, implementation science, mixed methods

## Abstract

**Background:**

Contact tracing is a vital public health tool used to prevent the spread of infectious diseases. However, traditional interview-format contact tracing (TCT) is labor-intensive and time-consuming and may be unsustainable for large-scale pandemics such as COVID-19.

**Objective:**

In this study, we aimed to address the limitations of TCT. The Yale School of Engineering developed a Hardware-Assisted Bluetooth-based Infection Tracking (HABIT) device. Following the successful implementation of HABIT in a university setting, this study sought to evaluate the performance and implementation of HABIT in a high school setting using an embedded mixed methods design.

**Methods:**

In this pilot implementation study, we first assessed the performance of HABIT using mock case simulations in which we compared contact tracing data collected from mock case interviews (TCT) versus Bluetooth devices (HABIT). For each method, we compared the number of close contacts identified and identification of unique contacts. We then conducted an embedded mixed methods evaluation of the implementation outcomes of HABIT devices using pre- and postimplementation quantitative surveys and qualitative focus group discussions with users and implementers according to the Reach, Effectiveness, Adoption, Implementation, and Maintenance framework.

**Results:**

In total, 17 students and staff completed mock case simulations in which 161 close contact interactions were detected by interview or Bluetooth devices. We detected significant differences in the number of close contacts detected by interview versus Bluetooth devices (*P*<.001), with most (127/161, 78.9%) contacts being reported by interview only. However, a significant number (26/161, 16.1%; *P*<.001) of contacts were uniquely identified by Bluetooth devices. The interface, ease of use, coherence, and appropriateness were highly rated by both faculty and students. HABIT provided emotional security to users. However, the prototype design and technical difficulties presented barriers to the uptake and sustained use of HABIT.

**Conclusions:**

Implementation of HABIT in a high school was impeded by technical difficulties leading to decreased engagement and adherence. Nonetheless, HABIT identified a significant number of unique contacts not reported by interview, indicating that electronic technologies may augment traditional contact tracing once user preferences are accommodated and technical glitches are overcome. Participants indicated a high degree of acceptance, citing emotional reassurance and a sense of security with the device.

## Introduction

### Background

Contact tracing is a key element of the public health response against the spread of infectious diseases [1-3]. Contact tracing implementation depends on a target pathogen’s characteristics and available treatments, vaccines, and other preventive services. It also frequently involves identifying, screening, and quarantining persons at risk of infection based on exposure to known cases, isolating and treating infected individuals, and later detecting other infected individuals. By tracing contacts, health professionals are able to document an individual’s risk and facilitate quarantine when necessary [3-7]. Its use has been highly successful in controlling the spread of tuberculosis, syphilis, Ebola virus, and SARS [1-3]. There is also evidence that contact tracing and accompanying isolation and quarantine likely blunted the emergence of the SARS-CoV-2 outbreak, containing the spread of virus in <4 months in the city of origin, Wuhan, China [3].

Traditional contact tracing is the status quo in the field of public health, typically managed by state, regional, and local health authorities. A traditional contact tracing strategy involves interviewing recently diagnosed cases and identifying with whom they have come into contact within specified physical distances, time frames, and lengths of each interaction. For example, a *close contact* for COVID-19 is defined per Centers for Disease Control and Prevention guidelines as someone within 6 feet (2 meters) of any case for at least 15 minutes over a 24-hour period [8,9]. This traditional method of contact tracing, although successful in many contexts, is labor-intensive and time-consuming, making it difficult to scale with an increasing number of SARS-CoV-2 infections [9]. Owing to these challenges, public health agencies struggled to trace COVID-19 contacts efficiently, especially in settings like schools and workplaces with high rates of contacts [10-12].

Schools have special social dynamics of concern for COVID-19 transmission including crowding in institutional environments and extracurricular activities that may increase risk of transmission [13,14]. Children and adolescents also have daily close contact with their parents, who may be active in many sectors of society. Thus, controlling outbreaks among youth helps keep schools safe and protects the well-being of the community at large [15-17]. Children and adolescents may struggle to follow guidance on mask use and hand hygiene and may have difficulty recalling their close contacts compared with adults, reducing the effectiveness of traditional contact tracing [18,19]. Given the potential impact of school-based contact tracing on reducing community transmission, we sought to increase the effectiveness of tracing within school settings with digital solutions based on suitable technologies.

After the onset of the COVID-19 pandemic, several app-based digital contact tracing methods have been proposed and implemented, though much remains unknown about their overall impact. Most contact tracing apps use GPS data or Bluetooth received signal strength indicator measurements as a proxy for distance. However, GPS data are only accurate up to an error of 10 to 15 feet; this is both insufficient for accurately detecting contacts within 6 ft and a poor indicator of location both in urban and in indoor settings [12,20,21]. Therefore, Bluetooth may be the most appropriate modality for measuring close contact interactions in school settings.

### Intervention—Hardware-Assisted Bluetooth-Based Infection Tracking

To address the limitations of traditional contact tracing and app-based digital contact tracing methods, such as scalability, notification delays, recall errors, and contact identification in public spaces, our colleagues at the Yale School of Engineering developed a carriable and wearable Hardware-Assisted Bluetooth-based Infection Tracking (HABIT) device that we previously pilot-tested in a university campus [22]. Implementation of the HABIT contact tracing intervention requires four components: (1) a local health center or clinic; (2) a central server; (3) carriable or wearable Bluetooth devices, called dongles thereafter; and (4) relay devices (smart phones or tablet) that transfer information from the dongles to the central server. These components are further described in the Multimedia Appendix 1.

### Prior Work

We previously compared this approach (without the encryption functionality active) with daily self-report interaction data in a university setting and found HABIT to have high sensitivity (94%) and specificity (95%) [23]. We also found this approach to be superior to a comparable app-based digital contact tracing tool developed by the university in terms of specificity, sensitivity, and usability. HABIT also addresses several privacy concerns related to other technology-assisted contact tracing interventions. As a local authority, the health center is permitted to know if any user has tested positive, but the system only allows the central server to gather limited interaction data about users who have interacted with COVID-19 cases. A user who never encounters a positive COVID-19 case remains anonymous to the central server. Furthermore, this approach does not require personal mobile devices to constantly emit and collect Bluetooth data, as this activity is restricted to the dongle. As such, HABIT is ideally suited for use in centralized organizations such as schools, universities, hospitals, or businesses in which every community member carries the dongle, and a local health authority is able to manage the system.

### Goals of the Study

Following the encouraging results from pilot implementation of HABIT in a controlled university environment, we wished to further evaluate its implementation in a real-world setting. Therefore, we implemented HABIT in a high school to test its performance and implementation success. We will refer to the independent prep school as “the School” for the remainder of this paper. In the first part of the paper, we evaluate the performance metrics of HABIT and subsequently examine implementation outcomes by integrating both quantitative and qualitative data. Our goal was to provide guidance through the results from this study to schools and other institutions seeking to implement digital contact tracing programs.

## Methods

### Study Design

**Figure 1:**
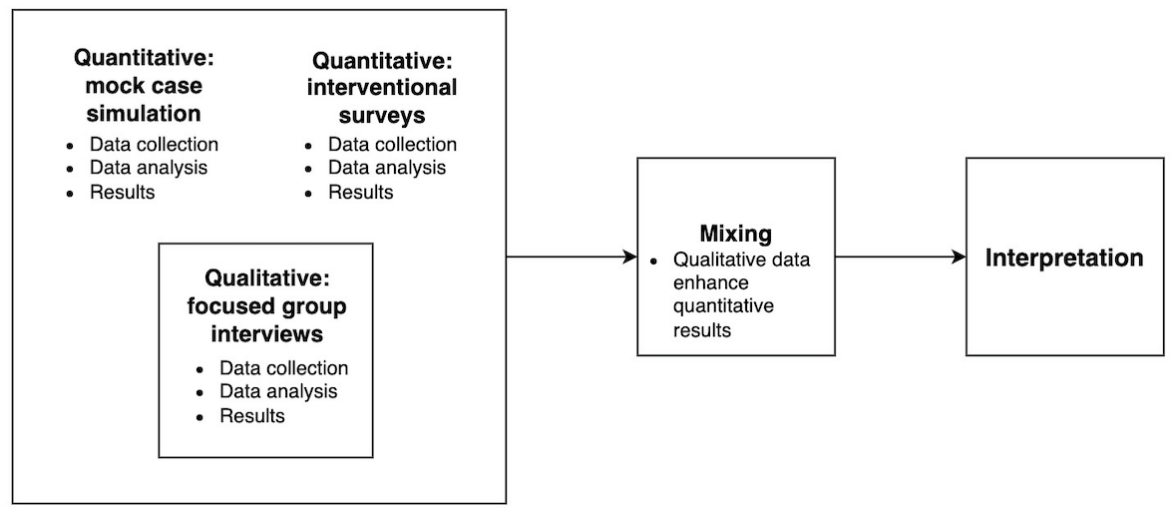
Embedded mixed methods design to examine the implementation of Hardware-Assisted Bluetooth-based Infection Tracking (HABIT).

We deployed an embedded mixed methods design (Figure 1) to evaluate the implementation of HABIT, combining externally valid insights from quantitative data with detailed contextual explanations from the qualitative data [24,25]. We first evaluated the performance metrics of HABIT as well as its implementation outcomes quantitatively through mock case simulations and survey analysis. Following the collection and analysis of the quantitative data, we conducted qualitative focus group discussions with 2 groups of participants to gain a deeper understanding of factors that affected the implementation of HABIT.

The complementary study design uses the qualitative data to clarify, illustrate, and provide more depth to the results from the quantitative log data to enhance interpretation and substantiate conclusions [26,27]. Results are synthesized through the aforementioned performance metrics and implementation outcomes using the Reach, Effectiveness, Adoption, Implementation, and Maintenance (RE-AIM) framework (Table 1) [28-30].

**Table 1:** Reach, Effectiveness, Adoption, Implementation, and Maintenance (RE-AIM) framework^a^.

RE-AIM framework component	Data collected	Experimental methods
Reach	Demographic characteristics	Intervention surveys
Effectiveness	Rates of contact identification by Bluetooth versus traditional methods	Mock case simulations
Adoption	Context	Focus Group
Implementation	Motivation, ease of use, user interface and satisfaction, usefulness, coherence, appropriateness, adherence, emotional impact, attitudes toward contact tracing	Intervention surveys Focus Group interview
Maintenance	Recommendation	Focus Group

^a^Implementation outcomes were selected based on the RE-AIM framework.

### Context

This study was conducted between March 2021 to May 2021. At the time of our study, the SARS-CoV-2 wild type (alpha strain) was the predominant strain. The Food and Drug Administration declared all adults in the United States (≥18 years) eligible for COVID-19 vaccines 1 week after our study began. All adolescents (ie, people aged 11-17 years) became eligible for vaccination 1 week before the end of our study period.

### School Site

The study was undertaken in collaboration with an independent school for girls in grades 6 through 12 in Northeast United States. There were approximately 300 students in the School, ranging in age from 11 to 19 years. The student-to-faculty ratio at the School is 7:1.

### Participants

Participant eligibility criteria included English fluency, ability to provide consent or assent, member of the School (students, teachers, or school staff), and at least 11 years old. We excluded middle school (grades 6-8) students from the mock case simulations and postintervention focus group interviews at the School’s request.

### Time Line

In December 2020, the research team began planning for this study. HABIT was tested by researchers before the start of the study to ensure that it was free of technical difficulties.

In February 2021, the research team and the School held a webinar for all faculty, students, and parents. The presentation covered a variety of topics, including the purpose of the digital contact tracing device, the development of HABIT and the dongle, the security features of HABIT, and the daily responsibilities of each study participant. The research team and a representative from the School responded to all questions from attendees. Following the webinar, a 20-page user manual was sent to the School health center and participants. The dongles were delivered to the School on March 18, 2021. The School organized and attached the dongles to lanyards and developed a distribution plan.

The School additionally described the study to all students again during a student assembly. Faculty advisers answered all questions during small group advising sessions with students. The consented participants were scheduled to pick up their devices between March 25, 2021, and April 1, 2021. The faculty adviser guided the participants through the process of setting up the app and syncing their devices. The School sent weekly reminders to the students to sync their HABIT devices. The teachers also reminded students verbally to adhere to the study protocol.

### Recruitment

#### General Study Participation

We emailed parents of all students to obtain opt-out parental permission for their children to participate in the study. The research team provided parents with the study information and parental permission forms. Parents had 2 weeks to opt out. At the end of the 2-week period, the research team and staff at the School distributed dongles to participants at the School. Student participants (regardless of age) completed electronic assent forms, and faculty or staff participants completed electronic consent forms at the time of dongle distribution. Participants were given freedom of consent or withdrawal throughout the study.

#### HABIT and Pre- and Postintervention Survey

All study participants were invited to complete pre- and postintervention surveys during dongle distribution and collection. Electronic assent and consent were embedded in the first page of the survey.

#### Mock Case Simulations

We randomly selected study participants in 2 batches of 30 without replacement through randomized number draws using R software (R Foundation for Statistical Computing). The sampling frame included all study participants who were staff or students in grades 9 to 12. We excluded randomly selected participants if for any reason they never received a dongle, never activated their dongle, were not present on campus during the period of interest or disclosed other personal reasons why they were unable to participate. We emailed selected participants with instructions to schedule a mock contact tracing interview. Randomly selected mock case interview participants were given US $10 gift cards upon completion of the second interview.

#### Focus Group Discussions

We selected students and staff from the School for focus group discussions based on their involvement in the study, interests in research participation, and perceived ability to speak about their experiences with Bluetooth contact tracing in a focus group setting. The School staff sent an opt-out parental permission request to the parents of selected students, giving parents at least 3 days to opt out. Following this period, the research team sent invitations to selected students and to adults, staff, and faculty at the School. Before the start of the focus group interviews, we collected consent through a survey via Qualtrics (Qualtrics). We used the Consolidated Criteria for Reporting Qualitative Research checklist to report the findings [31].

### Ethics Approval

This study was approved by the Institutional Review Board at Yale School of Public Health, Human Subjects Committee (#2000028753) and deemed to be minimal risk. We collected opt-out parental permission, student participants (regardless of age) assent, and faculty or staff participants consent. The consent and assent contained information about research study summary, benefits, risks, and freedom consent and withdrawal. We did not offer incentives for participation. Randomly selected mock case interview participants were given US $10 gift cards upon completion of the second interview. Their privacy were safeguarded through encryption of the Bluetooth-collected interaction data and secure storage of the web-app survey data. All data collected via interviews and surveys were anonymized at the earliest point possible to further protect their privacy.

### Data Collection and Analysis

#### Mock Case Simulations

The goal of this study component was to conduct mock contact tracing interviews with participants and subsequently compare their self-report interaction data with HABIT-collected data. We randomly selected study participants and invited them to complete a contact tracing interview and decrypt their HABIT data. We randomly selected a duration of 4 to 6 days extending backward from the date of interview invitation and considered this to be the time frame during which the mock case was “infectious” and able to “expose” other participants. We positioned this time period before the interview invitation assuming, for the sake of the simulation, that the interview would occur following “diagnosis and isolation” of the mock case. Of note, there is a potential risk for Bluetooth signals to be detected across walls, which would lead to identification of false-positive contacts. However, we discussed this risk extensively with our School partners who evaluated the physical layout of their classrooms and hallways and felt that it was unlikely for participants to be within 6 feet across walls owing to additional barriers such as tables, lockers, etc.

We conducted mock interviews via telephone and recorded data using Network Canvas (Complex Data Collective), a software for network data collection. We asked each participant to identify close contacts and describe the location and nature of the interaction (eg, classroom, eating together, sports, etc). We also asked participants to describe how often they carried their dongles and to identify any reasons why they did not carry the dongles while on campus. To analyze the data, we compared the number and list of close contacts identified by interview versus HABIT and tested 3 primary hypotheses using 2-tailed *t* tests (Table 2) to evaluate for differences between the contact lists. We excluded from the analyses (1) Bluetooth data from cases that did not complete the interview, (2) interview data from cases that did not decrypt their Bluetooth data, and (3) interview-reported contact interactions involving contacts that had not received a dongle.

**Table 2:** Mock case simulation hypothesis and results.

Null hypotheses	Equation	Statistical test	Accepted or rejected
No difference between the number of close contact interactions reported by interview versus Bluetooth	INT^a^-BT^b^=0	One-sample *t* test for significant deviation from “0”	Rejected (*P*<.001)
No unique interactions detected by interview	INT-D^c^=0	One-sample *t* test for significant deviation from “0”	Rejected (*P*<.001)
No unique interactions detected by Bluetooth	BT-D=0	One-sample *t* test for significant deviation from “0”	Rejected (*P*=.002)

^a^INT refers to the total number of close contact interactions reported by interview.

^b^BT refers to the total number of close contact interactions reported by Bluetooth (Hardware-Assisted Bluetooth-based Infection Tracking).

^c^D refers to the number of close contact interactions reported by Interview AND Bluetooth.

#### HABIT Sync Data

From the central server, we derived the number of times that participants synced their dongles within a 24-hour period. Although the central server cannot tell whether a participant synced several times a day, the syncing rate may serve as an approximate indicator of adherence. HABIT’s current memory capacity would approximately allow for storage of 1 week’s worth of contact data.

#### Pre- and Postintervention Surveys

The goal of this component of the study was to evaluate key implementation outcomes (ease of use, interface and satisfaction, acceptability, usefulness, coherence, setting, adherence, and appropriateness) quantitatively [27]. Data were collected via a web-based survey in English. We distributed the surveys to participants before they received their dongles and during dongle collection at the end of the study. We selected implementation outcomes based on the RE-AIM framework [29,30]. The preintervention survey included close-ended questions on demographic information, COVID-19 experiences, knowledge, and attitudes toward contact tracing and other public health interventions. The postintervention survey consisted of all aforementioned questions and closed-ended 5-point Likert scale questions on ease of use, interface and satisfaction, acceptability (Acceptability of Intervention Measure), usefulness (System Usability Scale), coherence, setting, adherence, appropriateness (Intervention Appropriateness Measures), and additional open-ended comments [27,29].

All statistical analyses were done in R software. Descriptive statistics were used to describe the central tendency and pattern of each variable. For descriptive purposes, categorical variables were expressed as proportions, whereas continuous variables were expressed as means with SDs. For the demographic variables, sample characteristics were summarized using descriptive statistics and compared across pre- and postinterventional surveys using 1-way independent analysis of variance and Pearson chi-square test, with statistical significance defined as *P*<.05. For all Likert scales for the key implementation outcomes, the score direction for each item was reversed when necessary to ensure that the directionality of the scores was consistent. Scores were reported as the percentage of respondents for each item. We calculated the score for each implementation outcome scale for every participant. The means and SDs for each score were reported. Internal consistency, a type of reliability, was measured using Cronbach α. For open-ended questions, we reviewed the responses and coded them for themes or patterns (see qualitative analysis description in the next section). We processed the data for both content and thematic analysis and used this information to contextualize the responses to the close-ended questions.

#### Focus Group Discussions

Two focus groups were conducted to gain an in-depth understanding of user experience and factors influencing HABIT implementation. The purpose of the first group, which consisted of 4 student participants, was to understand their experience and barriers and facilitators to participation and use of HABIT. The other focus group consisted of 4 faculty members and administrators with the aim of identifying difficulties and barriers associated with implementation. The faculty key stakeholders included individuals who are involved in the process of recruitment, distribution, identification, outreach, and education.

The structure of each session followed a semistructured interview design (Multimedia Appendix 2), following the Consolidated Criteria for Reporting Qualitative Research [31]. This approach was adopted to ensure an engaging and comprehensive discussion. A semistructured discussion guide (supplement) was developed according to the RE-AIM framework, with a particular focus on adoption, implementation, and maintenance [28,30] at both the setting and individual levels. Informed consents were signed before the interviews. The interview started with a brief explanation of the goal and content of the study. The domains of the interviews included (1) general experiences, (2) strengths and weaknesses of the implementation process, (3) barriers and unforeseen events, and (4) recommendations and improvements for sustainability. The focus groups were conducted via Zoom.

We analyzed the web-based focus groups through coding, categorizing, formulating themes, and connecting and interpreting them. A codebook was developed based on the RE-AIM framework for implementation research before transcription (DL, TS, and MB; Multimedia Appendix 3) [28,30]. It was further refined inductively during the coding phase. The research team (DL, TS, and MB) discussed and established codes in 3 meetings to maintain reflexivity. Focus group recordings were transcribed verbatim using Otter artificial intelligence and proofread by 2 independent researchers (DL and EE) for accuracy. The researchers reviewed the transcripts and coded the themes and patterns according to the codebook in Microsoft Word (Microsoft Corp; DL and TS). Conflicts were resolved by a third independent reviewer (MB). The code and categorization of themes were processed and summarized using Atlas TI (TS). We processed the data for both content and thematic analysis and used this information to contextualize the responses to the close-ended questions. The themes were organized according to the RE-AIM framework [28,30]. The results were summarized in paragraphs of continuous text and quotes.

## Results

### Sample Characteristics

Of the 304 school members, 284 (93.4%) members consented to participate in the study; 284 (93.4%) participants responded to the preintervention survey, and 112 (36.8%) responded to the postintervention survey. As presented in Table 3, in terms of gender, participants were mostly women (preintervention survey: 258/284, 90.8%; postintervention survey: 96/112, 85.7%) which is not surprising given that the School is an all-girls school. There were no statistically significant gender differences between pre- and postintervention surveys (*P*=.62). Racial and ethnic information were not collected from individuals because of the risk of exposing the identities of participants, given that most students at the School are White. For participants’ roles at the School, most (205/284, 72.2%) participants in the preintervention survey were students. There was an increasing percentage of faculty who filled out the postintervention survey (79/284, 27.8%) relative to the preintervention survey (51/112, 45.5%; *P*=.006). High school students (grades 9-12) represent most participants in both the pre- and postintervention surveys (182/205, 88.8% and 50/58, 86%, respectively).

In terms of living arrangements, approximately half (130/284, 45.8% and 45/112, 40.2%, pre- and postintervention survey, respectively) of the participants lived on campus and half (151/284, 53.2% and 66/112, 58.9%, pre- and postintervention survey, respectively) lived at home. Of the ones who lived on campus, most participants had 11 to 15 floormates (preintervention survey: 36/130, 27.7%; postintervention survey: 10/45, 22%). According to the preintervention survey, 38.5% (50/130) of participants had no roommate; 30.8% (40/130) had 1 roommate; 12.3% (16/130) had 2 roommates; 18.5% (24/130) had ≥3 roommates. According to the postintervention survey, 22% (10/45) of participants had no roommates; 18% (8/45) had 1 roommate; 27% (12/45) had 2 roommates; 33% (15/45) had ≥3 roommates. Participants had more roommates during the preintervention survey period compared with the postintervention period (*P*<.001). This observation can be explained by the continually evolving nature of the COVID-19 pandemic. Between the pre- and postinterventional periods, a number of breakthroughs were achieved regarding SARS-CoV-2 transmissions and COVID-19 prevention and treatment, which resulted in many changes to the safety protocol, such as the number of roommates. Of the ones who lived at home, most individuals have 3 or 4 family members (preintervention survey: 77/151, 51%; postintervention survey: 34/66, 52%; *P=*.24). In addition, 3.2% (9/151) and 15% (17/66) of individuals reported living with older adults (*P*=.26); and 20.1% (57/284) and 22.3% (25/112) reported living with people with chronic health conditions (*P*=.37) in pre- and postintervention surveys, respectively.

In total, 5.6% (16/284) of the preintervention survey respondents reported contracting SARS-CoV-2 before and during the study period. In the postintervention survey, 6.3% (7/112) of participants reported being infected with SARS-CoV-2. The extracurricular activities in order of participation were sports, music, academic club, and model United Nations. Overall, the characteristics of the postintervention survey were similar to those of the preintervention survey, with the exception of the percentage of faculty participation (*P*=.006) and the number of roommates (*P*<.001).

**Table 3:** Demographics of study participants.

		Preparticipation survey, (n=284), n (%)	Postparticipation survey, (n=112), n (%)	*P* value	
**Gender**					.62
	Women	258 (90.8)	96 (85.7)		
	Men	19 (6.7)	10 (8.9)		
	Prefer not to say	4 (1.4)	4 (3.6)		
	Prefer to self-describe	3 (1.1)	2 (1.8)		
**Role in the school**					.006
	Student	205 (72.2)	58 (51.8)		
	Faculty	79 (27.8)	51 (45.5)		
	Others	0 (0)	3 (2.7)		
**Grade**					.10
	6	5 (2.4)	3 (5)		
	7	9 (4.4)	5 (9)		
	8	9 (4.4)	0 (0)		
	9	33 (16.1)	15 (26)		
	10	59 (28.8)	12 (21)		
	11	49 (23.9)	14 (24)		
	12	41 (20)	9 (15)		
**Extracurricular activities^a^**					.99
	Sports	(42.2)	(42)		
	Dance	(6)	(8)		
	Music	(12.4)	(10.7)		
	Drama and play	(6.3)	(4.5)		
	Academic club	(11.5)	(11.6)		
	Model UN^b^	(11.5)	(11.6)		
	Student government	(5.7)	(6.3)		
	Others	(4.5)	(5.4)		
**Tested positive for COVID-19**					.76
	Positive for COVID-19	16 (5.6)	7 (6.3)		
**Living situation**					.69
	Campus	130 (45.8)	45 (40.2)		
	At home	151 (53.2)	66 (58.9)		
	Others	3 (1.1)	1 (0.9)		
**Number of roommates**					<.001
	None	50 (38.5)	10 (22)		
	1	40 (30.8)	8 (18)		
	2	16 (12.3)	12 (27)		
	≥3	24 (18.5)	15 (33)		
**Number of floor mate**					.12
	None	6 (4.6)	2 (4)		
	1-5	17 (13.1)	12 (27)		
	6-10	29 (22.3)	5 (11)		
	11-15	36 (27.7)	10 (22)		
	16-20	25 (19.2)	8 (18)		
	>20	17 (13.1)	8 (18)		
**Number of household member**					.24
	1-2	32 (21.2)	19 (28)		
	3-4	77 (51)	34 (52)		
	5-6	39 (25.8)	13 (20)		
	>6	3 (2)	0 (0)		
**Living with older adults**					.26
	Yes	9 (3.2)	17 (15)		
**Living with individuals with chronic conditions**					.37
	Yes	57 (20.1)	25 (22.3)		
	No	144 (50.7)	61 (54.5)		
	Not sure	82 (28.9)	23 (20.5)		
	Prefer not to say	1 (0.4)	3 (2.7)		

^a^Multiple selection question. Response is weighted.

^b^UN: United Nations.

### Mock Case Simulations

From May 3, 2021, to May 28, 2021, we randomly selected 60 participants from the School to invite for mock case interviews and subsequently excluded 10 (17%; 7/10, 70% never received nor initiated dongles; 3/10, 30% for other personal reasons). Of the remaining 83% (50/60) of mock cases, we interviewed 33 (66%; the remaining 17/50, 34% did not respond to the mock case interview requests). Out of 50 mock cases, 19 (38%) decrypted their Bluetooth data, 17 (89%) of whom were among those that had been interviewed. Approximately half were staff (9/17, 53%) and half were students (8/17, 47%) with roughly equal distribution across grades 9 to 12. Because the School is an all-women school, most (16/17, 94%) participants were women with only 1 man staff member.

Considering data from 17 mock cases that completed an interview and decrypted their HABIT data, we identified a total of 161 contact interactions involving 118 contacts. We found significant differences between the number of contacts reported by interview versus Bluetooth, and each method identified a significant number of unique contacts that were not identified by the opposing method (Table 2). Of the 161 interactions, we identified 127 (78.9%) by interview only, 26 (16.1%) by Bluetooth only, and 8 (5%) by both methods. Most (85/118, 72%) contacts were students with roughly equal distribution across grades 9-12 and only 7 (5.9%) contacts identified from grades 7 to 8.

### Interventional Outcomes—Implementation and Mechanism of Impact

#### Motivation and Emotional Impact

The focus group discussions revealed that participants were highly motivated and eager to use the HABIT device. The innovative approach sparked excitement and curiosity in the participants. One faculty member explained the following in the focus group interviews:

Our students were very eager, curious and excited at the prospect of getting this ‘blinky’ thing that they had to do something with.

The participants were primarily interested in using the device owing to a perceived increase in safety. Participants were reassured that the device would help them rapidly identify contacts in the event of an outbreak, as described by 1 student:

From a safety standpoint, it was reassuring to know while the study was going, that should we have any cases in the School,[...] we had this added degree of security as far as managing an outbreak.

An important benefit of the HABIT dongle is its effect on people’s emotional states. Most participants were very excited to use the HABIT dongle, as it provided a sense of security.

#### Attitudes Toward Contact Tracing

During the surveys, respondents were asked about their attitudes toward various methods of contact tracing (Multimedia Appendix 2). In general, people are willing to discuss recent activities (257/284, 90.5% and 95/112, 84.8%, pre- and postpartcipation survey, respectively) and contacts (250/284, 88% and 96/112, 85.7%, pre- and postpartcipation survey, respectively), but they are less likely to provide the names and phone numbers of their contacts (219/284, 77.1% and 95/112, 78.6%, pre- and postpartcipation survey, respectively), which may pose another barrier to the traditional interview-based contact tracing method. Most participants were willing to turn on Bluetooth for contact tracing (194/284, 68.3% and 69/112, 61.6%, pre- and postpartcipation survey, respectively), but fewer were hypothetically willing to turn on GPS for contact tracing owing to concerns over privacy (147/284, 51.8% and 63/112, 56.3%, pre- and postpartcipation survey, respectively). In the postintervention survey, participants reported using Bluetooth (29/112, 25.9%) more frequently than in the preintervention survey (38/284, 13.4%). The preintervention survey answers revealed that most (221/284, 77.8%) participants supported the use of school-owned contact tracing devices rather than those owned by the government. However, in the postintervention survey, more participants (39/284, 13.7% and 30/112, 26.8%; *P*<.001*,* pre- and postpartcipation survey, respectively) felt more neutral about the host of the contact tracing system, indicating that they are more comfortable with the concept of digital contact tracing.

#### Ease of HABIT Use

Most participants reported that they did not encounter any difficulty while installing and syncing the HABIT app (80/112, 71.4% and 84/112, 75%, respectively; Multimedia Appendix 4). As evident in the postintervention survey answers, many (84/112, 75%) found learning to use the syncing app quite easy and responded that carrying the device around school was convenient. Many factors, including webinars, informational sessions, and the student advisory group discussions, may have contributed to their satisfaction with the ease of use of the HABIT system.

#### User Interface and Satisfaction

Participants reported the syncing app provided information regarding their progress toward downloading and installing (64/112, 57.1%). The participants were neutral about the interface and organization of the syncing app (agree: 49/112, 44%; neutral: 24/112, 21.4%; disagree: 39/112, 34.6%). In general, most (56/112, 50%) participants were satisfied with the syncing system (Multimedia Appendix 5), as 1 student said the following in the focus group interviews:

Once you get on the app, it also [...] tells you what to do.

Most (51/112, 45.4%) participants would consider using the system again (Multimedia Appendix 5), as 1 student said the following:

I absolutely think I will

use the device in the future

#### Usefulness

Most (83/112, 73.8%) participants believed that this system would be useful for contact tracing. However, fewer (42/112, 37.3%) people believed that carrying the device improved their awareness of their social interactions. Some (52/112, 46.4%) people reported that the syncing app had all the functions in the compatibility it said it would have (Multimedia Appendix 6). However, some areas that needed to be worked on include the shape of the dongle and reminder notifications. Some students found the device to be “bulky,” making it difficult to carry regularly.

#### Coherence

Coherence was defined as participants’ understanding or misunderstanding of close contact definitions, how data are collected by the apps and dongles, how the data might be used for contact tracing, and how the systems protect their data. The survey respondents were largely (97/112, 86.9%) in agreement in understanding how the HABIT data would be used for contact tracing, illustrating that they felt that HABIT protected their privacy (83/112, 73.9%; Multimedia Appendix 7). The focus group participants elaborated on this point and commented on how they believed data collection was more consistent and “concrete” than traditional contact tracing interviews.

A high degree of coherence was achieved through educational sessions, as 1 faculty member described:

I spent a lot of time explaining the technology to adults and students in our community about [...] how Actually, it was very different than [...] apps.

#### Appropriateness

We defined “appropriateness” as “perceptions of the appropriateness of these technologies being used for contact tracing, including privacy concerns, downloading apps on personal phones, third parties, etc.” Most (90/112, 80.5%) of the participants believed that a contact tracing device would be appropriate for schools to use to more efficiently perform contact tracing. They (54/112, 48.3%) also preferred using a school-owned device as opposed to downloading an app on their personal phone (Multimedia Appendix 8).

However, many (52/112, 46%) individuals have concerns about how it could affect privacy. One faculty member elaborated the following:

I had just a couple of quick conversations with kids around not wanting their locations to be tracked.

Another reason the participants believed that this device was appropriate include the following:

There will always be those people who don’t get the vaccine and don’t care and won’t follow the rules. [...] I think it will always be a necessity to have like a backup plan and to have this type of security and the proper technology

#### Adherence

**Figure 2:**
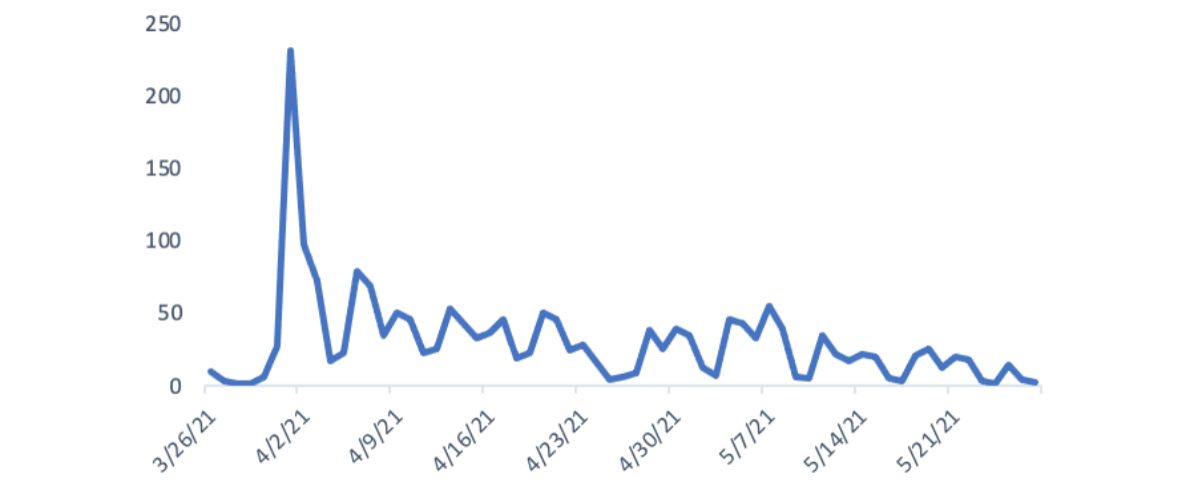
Adherence: daily syncing data extracted from Hardware-Assisted Bluetooth-based Infection Tracking (HABIT) central server.

Adherence was defined as the difficulty or ease of daily use of the devices (carrying, charging, remembering to use, etc), as well as self-reported adherence, methods of carrying the devices, and reasons for not carrying them. Through survey questionnaires, daily syncing data, focus group interviews, and mock case interviews, we assessed the degree to which HABIT devices were paired, synced, and used (Multimedia Appendix 9). Adherence is one of the most challenging barriers to the implementation of the HABIT device. In total, 64% (21/33) of participants from the mock case interview reported carrying their devices with them constantly, whereas 30% (10/33) of them left it out of reach for 2 days before the interview. The syncing rate peaked at the beginning of study but gradually decreased as the study progressed (Figure 2). The survey questionnaire and focus group interview provided possible reasons for the poor and decreasing adherence as the research continued.

Certain design issues of HABIT may have reduced adherence and prevented students from integrating it into their daily routines. As 1 student explained:

I didn’t really know what to do with it while playing sports.

Some participants mentioned how an added device was a major reason that many stopped carrying the device. In addition, 57% (19/33) of the respondents reported that forgetting the device at home was the reason for poor adherence:

From a logistical standpoint, and a practical use standpoint, I think those were the big challenges were just one more thing to charge or keep a hold of or recall that you even have.

#### Technical Difficulty and Recommendations

Several technical difficulties adversely impacted the device’s usability and adherence. The technical difficulties can be divided into 2 categories.

First, technical challenges during syncing were one of the reasons that many participants stopped syncing the device as the study continued:

If you forgot to sync for a day, there was so much data that it just took so long to do it.

Second, the brightness of the light on the dongle reduced the satisfaction for some participants:

I remember people specifically talking about the brightness of the light, and how light prevented some of them from sleeping. [...] For such a small device, it emits a large amount of light.

Some participants suggested adding a notification feature to facilitate adherence to syncing and use:

If the app that it was on had a reminder...like maybe at a certain time at night that [...] “Remember to charge it” or in the morning like “remember to bring it with you” because that was like something I always forgot...

Furthermore, 92.9% (104/112) of the participants in the survey questionnaire said that they would be more likely to carry a device around if it were smaller than the 1 used in the pilot study (Multimedia Appendix 9). One participant said during the focus group discussions that if the shape of the device was modeled to fit in their hands, then they would be more likely to carry it around.

## Discussion

### Principal Findings

Contact tracing is one of the most fundamental public health strategies for infectious disease control. Despite its utility and documented effectiveness in multiple disease settings [23], there is a paucity of research on the effectiveness and usability of digital contact tracing systems. Most studies that assess the effectiveness of digital contact tracing methods are modeling studies. Modeling studies reflect considerable uncertainty, thus it is difficult to objectively assess how realistic the assumptions (and therefore the results) of modeling studies are [32]. Among the few studies where digital contact tracing has been tested, implementation outcomes and user acceptability have not been addressed [33,34]. Our study is the first to examine the performance and implementation of a novel contact tracing method in a secondary school.

In this implementation study, participants found the technology to be appropriate for school-based contact tracing and were eager to participate and use the novel HABIT device. Syncing difficulties, however, significantly reduced the usability of the technology and led to decreased engagement over time. Despite these barriers, HABIT was still able to identify unique close contact interactions that were not reported during traditional interviews, pointing to the potential benefit of using HABIT or similar technology in school or institutional settings to enhance contact tracing. We also expect that the performance of this study device may improve once we address the syncing difficulties and add reminder features to the app for better adherence. Despite the setbacks, participants reported that HABIT provided a sense of security, which can be extremely beneficial to students’ mental well-being during a pandemic.

This study builds upon an earlier pilot study evaluating HABIT on a college campus and is one of the first mixed methods studies to evaluate such a tool for COVID-19 contact tracing in a school setting. Compared with our previous college campus study, which reported high sensitivity (94%) and specificity (95%), this study reported decreased performance when HABIT was compared with traditional interview methods in a less-controlled high school setting, partially owing to technical difficulties and poor adherence, which should be addressed in the future implementation studies and investigated further. School safety is an urgent topic currently because of the debates over school openings and continuing prevention measures in the context of new SARS-CoV-2 variants. Implementing interventions among teenagers within a school setting is one of the most challenging scenarios, as teenage adherence to many interventions is 40% lower than that of adults [35]. Hence, our study taps into these challenges and provides valuable insights into the implementation of digital contact tracing in a “real world” setting.

Overall, the faculty and students had high satisfaction with the interface, ease of use, coherence, and appropriateness. However, the shape and design of the device can be improved to increase user adherence and usability. In addition to addressing the technical difficulties, we recommend that the app provide syncing and charging reminders to increase usability. Many found that HABIT provides a sense of security, which can be extremely beneficial during a pandemic. Further research is needed to determine implementation strategies for improving adherence and therefore maximizing HABIT’s performance. The study points to the necessity to use digital contact tracing and provides valuable guidance on implementing contact tracing interventions in a school setting. Contact tracing can be incredibly useful to keep students, teachers, and other school members safe and healthy in the face of global pandemic or outbreaks.

### Strengths and Limitations

There are several notable strengths of this study. First, this study was conducted in a real-world high school setting, allowing for broader generalizability of our findings. Second, our use of mixed methods allows for triangulation of data and increases the internal validity of our findings. Third, the study is timely in offering information about contact tracing in secondary schools. The importance and originality of the intervention advance the understanding of implementing digital contact tracing in secondary schools.

There are also several limitations to this study. First, the School was composed of mostly women and White participants, and we were not able to collect any racial and ethnic information owing to the risk of exposing participants’ identifies. This therefore limits the generalizability of our findings. However, other studies on adolescent medication adherence have identified similar types of implementation difficulties, for example, low adherence, especially around exam periods; stress; etc [36,37]. This implies that our findings likely remain relevant in other types of high schools in the United States. Second, the participation rate for our survey questionnaire dropped 40% in the postintervention survey. The low response rate to the postintervention survey may lead to response bias and may not represent the entire target population. Despite the low participation rate, we were able to conduct planned statistical analyses and identify significant findings despite the reduction in power. Third, our study was also limited by recall bias because participants were asked to recall events 2 months after they occurred with self-reported data collected in the focus group discussions and the postintervention survey. Last, it is uncertain if all close contacts reported by interview and Bluetooth truly met the criteria for close contact interactions (within 6 feet for at least 15 minutes). We initially intended to conduct follow-up interviews with each mock case to discuss discrepancies between the interview and Bluetooth contact lists but were ultimately unable to complete this activity owing to logistical limitations. Despite these limitations, this study serves as a starting point for the implementation of contact tracing devices in primary and secondary school settings.

### Conclusions

Despite the increasing use of digital contact tracing in the mitigation of pandemics, there is a lack of empirical evidence of its effectiveness and usability. This study aims to address this gap in the literature by implementing a “HABIT” system in a challenging secondary school setting during the COVID-19 pandemic. The effectiveness of the HABIT in picking up contacts that were not reported through traditional interview-format contact tracing confirms that more efforts should be made to promote optimized development and implementation of HABIT during a respiratory viral pandemic. An embedded mixed methods design was used to collect and analyze data from students and staff to determine the feasibility and usefulness of such an intervention. Both faculty and students were very satisfied with the interface, ease of use, coherence, and appropriateness. We identified some important challenges in the implementation process. The study showed that the technical components and adherence were suboptimal, leading to suboptimal user experiences and reduced performance. Thus, more attention should be paid to improving technical usability and exploring strategies for improving adherence. Findings of this study will be useful in addressing implementation strategies among adolescents in a secondary school setting, enhancing implementation success. Use of HABIT and strategies to ensure adherence appear to be pivotal. The prototype design and technical difficulties during syncing can be improved to increase usability and adherence. Further studies addressing these factors are needed. The added degree of emotional reassurance and sense of security provided by digital contact tracing should also be explored further. During a respiratory viral pandemic, Bluetooth digital contact tracing can play a critical role in keeping school members safe.

## Appendix

Multimedia Appendix 1 Description of Hardware-Assisted Bluetooth-based Infection Tracking.

Multimedia Appendix 2 Attitudes toward contact tracing. Data on attitudes toward contact tracing were collected through a questionnaire in the postintervention survey.

Multimedia Appendix 3 Codebook.

Multimedia Appendix 4 Ease of use data were collected through a 5-point Likert scale questionnaire in the postintervention survey.

Multimedia Appendix 5 Interface and satisfaction were collected through a 5-point Likert scale questionnaire in the postintervention survey.

Multimedia Appendix 6 Usefulness data were collected through a 5-point Likert scale questionnaire in the postintervention survey.

Multimedia Appendix 7 Coherence data were collected through a 5-point Likert scale questionnaire in the postintervention survey.

Multimedia Appendix 8 Appropriateness data were collected through a 5-point Likert scale questionnaire in the postintervention survey.

Multimedia Appendix 9 Adherence.

## Abbreviations

HABIT: Hardware-Assisted Bluetooth-based Infection Tracking
RE-AIM: Reach, Effectiveness, Adoption, Implementation, and Maintenance
TCT: traditional interview-format contact tracing

## Data Availability

Our raw data are available upon requesting the corresponding author.
