# Supplementary material for: Implementation of a Hardware-Assisted Bluetooth-Based COVID-19 Tracking Device in a High School: Mixed Methods Study
Source: JMIR Form Res. 2023 Apr 7;7:e39765. doi: 10.2196/39765 (PMC10131711; doi:10.2196/39765)
Supplement: Multimedia Appendix 1 [file formative_v7i1e39765_app1.docx]

**Supplement**

**Supplemental Text**

HABIT Details

The local health center is responsible for the distribution of Bluetooth dongles to users, managing data stored within the central server, administering testing when appropriate, and notifying “close contacts” of any potential exposures to positive cases. The Bluetooth dongle is small (20 grams; 6cm*3cm*1.5cm; see Supplemental Figure 2) and can be easily carried on a keyring or lanyard similar to commonly used electronic automobile fobs. Each Bluetooth dongle has a unique serial ID linking the dongle to its user. The dongles transmit a separate ID (“Dongle ID”) which is randomly generated by the dongle and refreshed every hour to prevent user tracking. Each dongle records signals emitted by other HABIT dongles, documenting the distance between dongles (based upon signal strength) and the duration of each interaction. Interaction data as well as the receiver dongle’s serial ID is encrypted and stored locally until the dongle is “synced” with a relay device and data are transferred to the central server.

Privacy is guaranteed with advanced cybersecurity protections (Supplemental Figure 3). The central server stores a list of all serial IDs but, for user security, is unable to discern which Dongle IDs belong to which dongles and users. The interaction data is encrypted using the received Dongle ID as the key. The decryption of this data relies on multiple steps, beginning with the identification of a positive COVID-19 case. When a case is identified, the local health center provides the case with a “decryption token” that can be entered into the relay device. During the next syncing event, the dongle will provide the central server with the list of all Dongle IDs emitted by the dongle during the case’s infectious period. When any close contacts sync their data, the central server will then be able to use the case’s Dongle IDs to decrypt the close contact’s interaction data, and subsequently discover the Serial ID of the close contact. In summary, HABIT protects the privacy of users as much as possible. If a user is never close to a positive COVID-19 case, the user’s identity and interaction information are encrypted and hidden from all other users, the health center and the central server. Data is decrypted only when a case is found and only for close contacts of the case. The rest of the data remains encrypted. For a close contact, the health center only knows the identity of the close contact and all the positive cases he/she was in contact with, but nothing else.

**Supplemental Figures**


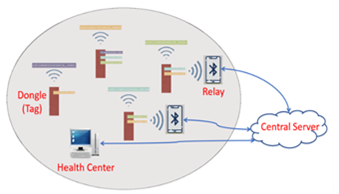


***Supplemental Figure 1.* *Hardware-Assisted Bluetooth-based Infection Tracking Operation Components.*** The implementation of HABIT requires four components: 1) a local health center, 2) a central server, 3) carriable/wearable Bluetooth dongles, and 4) relay devices (phones or tablets) that transfer information from the dongles to the central server.


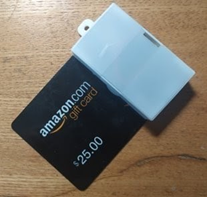


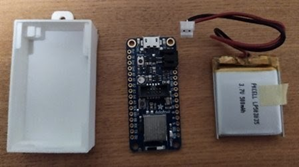


***Supplemental Figure 2. HABIT Hardware Dongle.***

Figure 2A shows the dimension of the dongle. The dongle is small, weighing 20 grams. The size of the dongle is 6cm*3cm*1.5cm. Figure 2B shows the assembly of the hardware dongle.


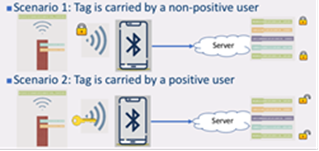


***Supplemental Figure 3. HABIT Security and Privacy****.* Multistep decryption process ensures the security of user privacy.

***Supplemental Table 1. Attitudes toward contact tracing.*** Data on attitudes toward contact tracing were collected through a questionnaire in the pre- and post-participation survey.

|  | Preparticipation n (%), N = 284 | Postparticipation n (%), N = 112 | P-Values |
| --- | --- | --- | --- |
| Willingness to Discuss Recent Activities | | | *P = .28* |
| Yes | 257 (90.5%) | 95 (84.8%) |  |
| No | 4 (1.4%) | 6 (5.4%) |  |
| I am not sure | 23 (8.1%) | 11 (9.8%) |  |
| Willingness to Discuss Contacts | | | *P = .21* |
| Yes | 250 (88.0%) | 96 (85.7%) |  |
| No | 6 (2.1%) | 8 (7.1%) |  |
| I am not sure | 28 (9.9%) | 8 (7.1%) |  |
| Willingness to Provide Contact Names | | | *P = .91* |
| Yes | 219 (77.1%) | 88 (78.6%) |  |
| No | 13 (4.6%) | 4 (3.6%) |  |
| I am not sure | 52 (18.3%) | 20 (17.9%) |  |
| Willingness to Provide Contact Phone Number | | | *P = .99* |
| Yes | 131 (46.1%) | 51 (45.5%) |  |
| No | 38 (13.4%) | 16 (14.3%) |  |
| I am not sure | 115 (40.5%) | 45 (40.2%) |  |
| Frequency of Bluetooth Usage | | | *P = .02* |
| Keep it on all time | 38 (13.4%) | 29 (25.9%) |  |
| Everyday | 149 (52.5%) | 49 (43.8%) |  |
| Most Days | 51 (18.0%) | 12 (10.7%) |  |
| Some Days | 21 (7.4%) | 19 (17.0%) |  |
| Rarely | 20 (7.0%) | 3 (2.7%) |  |
| Never | 5 (1.8%) | 0 (0.0%) |  |
| Willingness to turn on Bluetooth for Contact Tracing | | | *P = .38* |
| Yes, all the time | 194 (68.3%) | 69 (61.6%) |  |
| Yes, when I am on campus | 59 (20.8%) | 31 (27.7%) |  |
| Yes, only under special circumstances | 3 (1.1%) | 0 (0.0%) |  |
| No | 5 (1.8%) | 5 (4.5%) |  |
| I am not sure | 23 (8.1%) | 7 (6.3%) |  |
| Willingness to turn on GPS for Contact Tracing | | | *P = .61* |
| Yes | 147 (51.8%) | 63 (56.3%) |  |
| No | 49 (17.3%) | 21 (18.8%) |  |
| I am not sure | 85 (29.9%) | 28 (25.0%) |  |
| Not Applicable | 3 (1.1%) | 0 (0.0%) |  |
| Prefer School-Owned Contact Tracing | | | *P < 0.001* |
| Strongly Agree | 99 (34.9%) | 31 (27.7%) |  |
| Moderately Agree | 86 (30.3%) | 28 (25.0%) |  |
| Slightly Agree | 36 (12.7%) | 19 (17.0%) |  |
| Neutral | 39 (13.7%) | 30 (26.8%) |  |
| Slightly Disagree | 21 (7.4%) | 0 (0.0%) |  |
| Moderately Disagree | 3 (1.1%) | 4 (3.6%) |  |
| Strongly Disagree | 0 (0.0%) | 0 (0.0%) |  |
| I have concerns about privacy | | | *P = .52* |
| Strongly Agree | 38 (13.4%) | 8 (7.1%) |  |
| Moderately Agree | 72 (25.4%) | 27 (24.1%) |  |
| Slightly Agree | 56 (19.7%) | 27 (24.1%) |  |
| Neutral | 72 (25.4%) | 26 (23.2%) |  |
| Slightly Disagree | 9 (3.2%) | 9 (8.0%) |  |
| Moderately Disagree | 17 (6.0%) | 10 (8.9%) |  |
| Strongly Disagree | 20 (7.0%) | 5 (4.5%) |  |

***Supplemental Table 2.* Codebook*.***


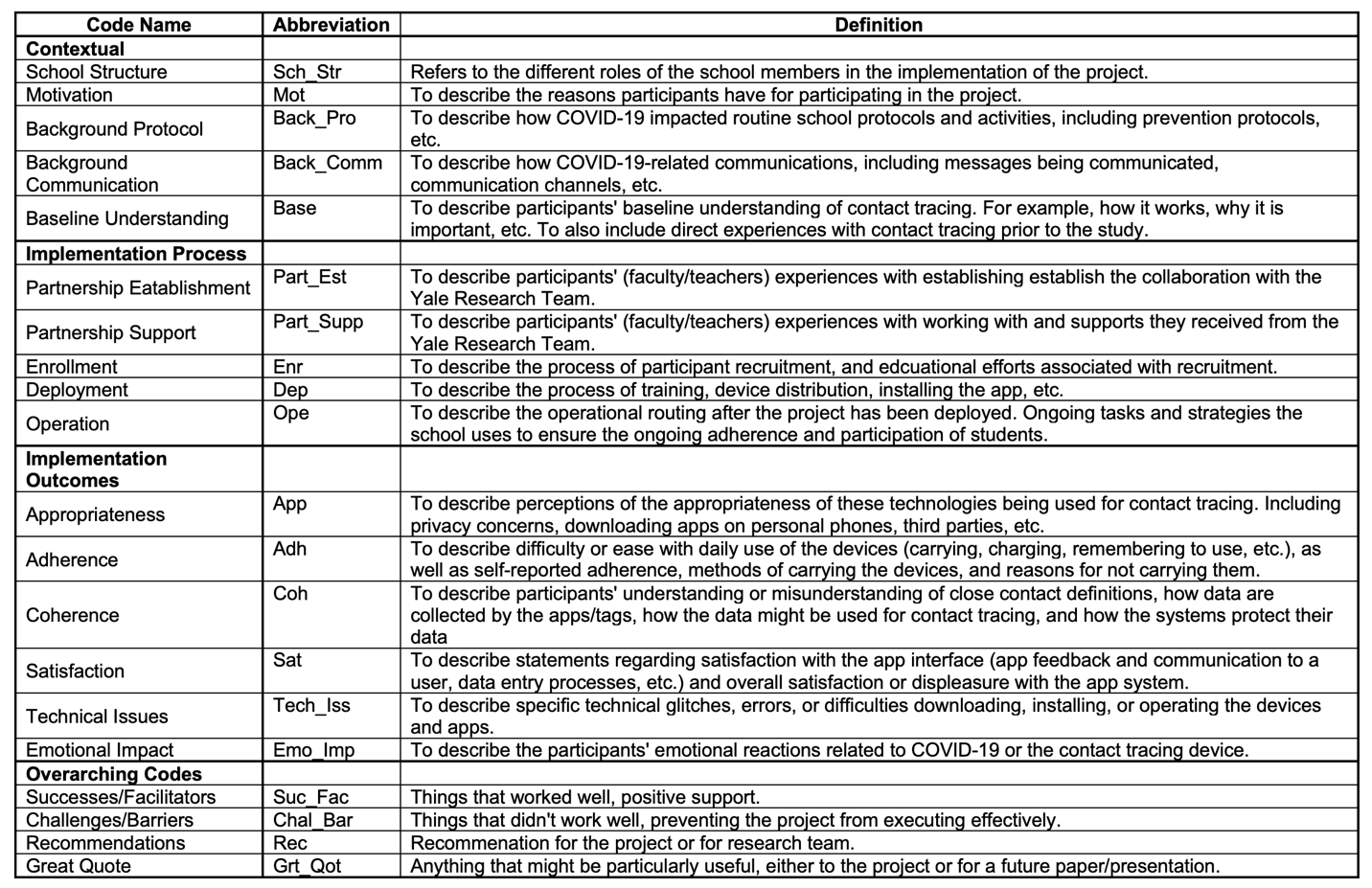


***Supplemental Table 3.* Ease of Use*.*** Ease of use data were collected through a 5-point Likert scale questionnaire in the postintervention survey.

| Ease of Use | Agree n (%) | Neutral n (%) | Disagree n (%) |
| --- | --- | --- | --- |
| It was easy for me to install the syncing app on the device | 80 (71.4%) | 3 (2.4%) | 29 (26.2%) |
| It was easy for me to learn to use the syncing app | 84 (75.0%) | 9 (8.3%) | 19 (16.7%) |
| The syncing app was easy to use | 68 (60.7) | 5 (4.8%) | 39 (34.5%) |
| The device was convenient to carry with me throughout my school day/while on the school campus | 61 (54.8) | 8 (7.1%) | 43 (38.1%) |

***Supplemental Table 4.* Interface and Satisfaction*.*** Interface and satisfaction data were collected through a 5-point Likert scale questionnaire in the postintervention survey.

| Interface and Satisfaction | Agree n (%) | Neutral n (%) | Disagree n (%) |
| --- | --- | --- | --- |
| I like the interface of the syncing app | 36 (32.2%) | 33 (29.8%) | 43 (38.0%) |
| The information in the syncing app was well organized, so I could easily find the information I needed | 49 (44.0%) | 24 (21.4%) | 39 (34.6%) |
| The syncing app adequately acknowledged and provided information to let me know the progress of my action | 64 (57.1%) | 17 (15.5%) | 31 (27.4%) |
| The amount of time involved in using the syncing app is acceptable | 63 (55.9%) | 9 (8.3%) | 40 (35.8%) |
| I would use the device and syncing app again | 51 (45.4%) | 21 (19.0%) | 40 (35.6%) |
| Overall, I am satisfied with this system | 56 (50%) | 21 (19.0%) | 35 (31.0%) |

***Supplemental Table 5.* Usefulness*.*** Usefulness data were collected through a 5-point Likert scale questionnaire in the postintervention survey.

| Usefulness | Agree n (%) | Neutral n (%) | Disagree n (%) |
| --- | --- | --- | --- |
| The system would be useful for contact tracing | 83 (73.8%) | 12 (10.7%) | 17 (15.5%) |
| Carrying the device with me helped increased my awareness of my social interactions | 42 (37.3%) | 20 (18.1%) | 50 (44.6%) |
| The syncing app has all the functions and capabilities I expected it to have | 52 (46.4%) | 35 (31.0%) | 25 (22.6%) |

***Supplemental Table 6.* Coherence*.*** Coherence data were collected through a 5-point Likert scale questionnaire in the postintervention survey.

| Coherence | Agree n (%) | Neutral n (%) | Disagree n (%) |
| --- | --- | --- | --- |
| I understand how data collected with this system would be used for contact tracing | 97 (86.9%) | 7 (6.0%) | 8 (7.1%) |
| I understand how this system currently protects my privacy | 83 (73.9%) | 15 (13.1%) | 15 (13.0%) |

***Supplemental Table 7.* Appropriateness*.*** Appropriateness data were collected through a 5-point Likert scale questionnaire in the postintervention survey.

| Appropriateness | Agree n (%) | Neutral n (%) | Disagree n (%) |
| --- | --- | --- | --- |
| It is appropriate for your school to use Bluetooth devices to monitor interactions on campus in order to more efficiently perform contact tracing | 90 (80.5%) | 14 (12.2%) | 8 (7.3%) |
| It is appropriate to use location information such as GPS and/or Wi-Fi connection data for contact Tracing | 67 (60.3%) | 18 (15.7%) | 27 (24.1%) |
| I would prefer to use a school-owned contact tracing device as opposed to downloading an app on my personal phone | 54 (48.3%) | 35 (31.0%) | 23 (20.7%) |
| I have concerns about how using this system, or a system like it, could affect my privacy | 52 (46.0%) | 21 (19.0%) | 39 (35.0%) |

***Supplemental Table 8.* Adherence*.***

| Table S8A. | Adherence | | Agree n (%) | | Neutral n (%) | Disagree n (%) |
| --- | --- | --- | --- | --- | --- | --- |
|  | Would carry if larger | | 1 (0.9%) | | 8 (7.1%) | 103 (93.6%) |
|  | Would carry if smaller | | 104 (92.9%) | | 7 (6.3%) | 3 (2.7%) |
|  |  | |  | |  |  |
| Table S8B. | What prevents you from carrying the device? | | n (%) | |  |  |
|  | Forgot at home/dorm | | 19 (57%) | |  |  |
|  | Forgot at Desk | | 6 (18%) | |  |  |
|  | Intentionally left at home/dorm | | 2 (6%) | |  |  |
|  | Intentionally left at desk or in locker | | 1 (3%) | |  |  |
|  | Others | | 5 (15%) | |  |  |
|  |  | |  | |  |  |
|  |  | |  | |  |  |
| Table S8C. | How did you carry the device with you throughout the day? | | n % | |  |  |
|  | Pocket | | 9 (28%) | |  |  |
|  | Backpack/Purse | | 8 (24%) | |  |  |
|  | Attached to a lanyard or belt | | 6 (18%) | |  |  |
|  | Attached to my clothes | | 4 (12%) | |  |  |
|  | Left on school desk | | 2 (6%) | |  |  |
|  | Left in the dorm | | 2 (6%) | |  |  |
|  | Left at home | | 1 (3%) | |  |  |
|  | Others | | 1 (3%) | |  |  |
|  |  | |  | |  |  |
|  |  | |  | |  |  |
| Table S8D. | For what proportion (%) of your total school days did you have the device either on you or within arms' reach? | | n % | |  |  |
|  | 100% | | 6 (18%) | |  |  |
|  | 99-95% | | 4 (12%) | |  |  |
|  | 94-90% | | 3 (10%) | |  |  |
|  | 89-85% | | 4 (12%) | |  |  |
|  | 84-80% | | 2 (6%) | |  |  |
|  | 79-75% | | 2 (6%) | |  |  |
|  | 74-70% | | 2 (6%) | |  |  |
|  | 69-65% | | 1 (3%) | |  |  |
|  | 64-60% | | 0 (0%) | |  |  |
|  | 59-55% | | 2 (6%) | |  |  |
|  | 54-50% | | 1 (3%) | |  |  |
|  | below 50% | | 6 (18%) | |  |  |
|  |  |  | |  |  |  |
