# Supplementary material for: Implementation of a Hardware-Assisted Bluetooth-Based COVID-19 Tracking Device in a High School: Mixed Methods Study
Source: JMIR Form Res. 2023 Apr 7;7:e39765. doi: 10.2196/39765 (PMC10131711; doi:10.2196/39765)
Supplement: Multimedia Appendix 2 [file formative_v7i1e39765_app2.docx]

|  | Preparticipation n (%), N = 284 | Postparticipation n (%), N = 112 | P-Values |
| --- | --- | --- | --- |
| Willingness to Discuss Recent Activities | | | *P = .28* |
| Yes | 257 (90.5%) | 95 (84.8%) |  |
| No | 4 (1.4%) | 6 (5.4%) |  |
| I am not sure | 23 (8.1%) | 11 (9.8%) |  |
| Willingness to Discuss Contacts | | | *P = .21* |
| Yes | 250 (88.0%) | 96 (85.7%) |  |
| No | 6 (2.1%) | 8 (7.1%) |  |
| I am not sure | 28 (9.9%) | 8 (7.1%) |  |
| Willingness to Provide Contact Names | | | *P = .91* |
| Yes | 219 (77.1%) | 88 (78.6%) |  |
| No | 13 (4.6%) | 4 (3.6%) |  |
| I am not sure | 52 (18.3%) | 20 (17.9%) |  |
| Willingness to Provide Contact Phone Number | | | *P = .99* |
| Yes | 131 (46.1%) | 51 (45.5%) |  |
| No | 38 (13.4%) | 16 (14.3%) |  |
| I am not sure | 115 (40.5%) | 45 (40.2%) |  |
| Frequency of Bluetooth Usage | | | *P = .02* |
| Keep it on all time | 38 (13.4%) | 29 (25.9%) |  |
| Everyday | 149 (52.5%) | 49 (43.8%) |  |
| Most Days | 51 (18.0%) | 12 (10.7%) |  |
| Some Days | 21 (7.4%) | 19 (17.0%) |  |
| Rarely | 20 (7.0%) | 3 (2.7%) |  |
| Never | 5 (1.8%) | 0 (0.0%) |  |
| Willingness to turn on Bluetooth for Contact Tracing | | | *P = .38* |
| Yes, all the time | 194 (68.3%) | 69 (61.6%) |  |
| Yes, when I am on campus | 59 (20.8%) | 31 (27.7%) |  |
| Yes, only under special circumstances | 3 (1.1%) | 0 (0.0%) |  |
| No | 5 (1.8%) | 5 (4.5%) |  |
| I am not sure | 23 (8.1%) | 7 (6.3%) |  |
| Willingness to turn on GPS for Contact Tracing | | | *P = .61* |
| Yes | 147 (51.8%) | 63 (56.3%) |  |
| No | 49 (17.3%) | 21 (18.8%) |  |
| I am not sure | 85 (29.9%) | 28 (25.0%) |  |
| Not Applicable | 3 (1.1%) | 0 (0.0%) |  |
| Prefer School-Owned Contact Tracing | | | *P < 0.001* |
| Strongly Agree | 99 (34.9%) | 31 (27.7%) |  |
| Moderately Agree | 86 (30.3%) | 28 (25.0%) |  |
| Slightly Agree | 36 (12.7%) | 19 (17.0%) |  |
| Neutral | 39 (13.7%) | 30 (26.8%) |  |
| Slightly Disagree | 21 (7.4%) | 0 (0.0%) |  |
| Moderately Disagree | 3 (1.1%) | 4 (3.6%) |  |
| Strongly Disagree | 0 (0.0%) | 0 (0.0%) |  |
| I have concerns about privacy | | | *P = .52* |
| Strongly Agree | 38 (13.4%) | 8 (7.1%) |  |
| Moderately Agree | 72 (25.4%) | 27 (24.1%) |  |
| Slightly Agree | 56 (19.7%) | 27 (24.1%) |  |
| Neutral | 72 (25.4%) | 26 (23.2%) |  |
| Slightly Disagree | 9 (3.2%) | 9 (8.0%) |  |
| Moderately Disagree | 17 (6.0%) | 10 (8.9%) |  |
| Strongly Disagree | 20 (7.0%) | 5 (4.5%) |  |
