# Supplementary material for: Implementation of a Hardware-Assisted Bluetooth-Based COVID-19 Tracking Device in a High School: Mixed Methods Study
Source: JMIR Form Res. 2023 Apr 7;7:e39765. doi: 10.2196/39765 (PMC10131711; doi:10.2196/39765)
Supplement: Multimedia Appendix 3 [file formative_v7i1e39765_app3.pdf]

Code Name	Abbreviation	Definition
**Contextual**		
School Structure	Sch_Str	Refers to the different roles of the school members in the implementation of the project.
Motivation	Mot	To describe the reasons participants have for participating in the project.
Background Protocol	Back_Pro	To describe how COVID-19 impacted routine school protocols and activities, including prevention protocols, etc.
Background Communication	Back_Comm	To describe how COVID-19-related communications, including messages being communicated, communication channels, etc.
Baseline Understanding	Base	To describe participants' baseline understanding of contact tracing. For example, how it works, why it is important, etc. To also include direct experiences with contact tracing prior to the study.
**Implementation Process**		
Partnership Eatablishment	Part_Est	To describe participants' (faculty/teachers) experiences with establishing establish the collaboration with the Yale Research Team.
Partnership Support	Part_Supp	To describe participants' (faculty/teachers) experiences with working with and supports they received from the Yale Research Team.
Enrollment	Enr	To describe the process of participant recruitment, and edcuational efforts associated with recruitment.
Deployment	Dep	To describe the process of training, device distribution, installing the app, etc.
Operation	Ope	To describe the operational routing after the project has been deployed. Ongoing tasks and strategies the school uses to ensure the ongoing adherence and participation of students.
**Implementation Outcomes**		
Appropriateness	App	To describe perceptions of the appropriateness of these technologies being used for contact tracing. Including privacy concerns, downloading apps on personal phones, third parties, etc.
Adherence	Adh	To describe difficulty or ease with daily use of the devices (carrying, charging, remembering to use, etc.), as well as self-reported adherence, methods of carrying the devices, and reasons for not carrying them.
Coherence	Coh	To describe participants' understanding or misunderstanding of close contact definitions, how data are collected by the apps/tags, how the data might be used for contact tracing, and how the systems protect their data
Satisfaction	Sat	To describe statements regarding satisfaction with the app interface (app feedback and communication to a user, data entry processes, etc.) and overall satisfaction or displeasure with the app system.
Technical Issues	Tech_Iss	To describe specific technical glitches, errors, or difficulties downloading, installing, or operating the devices and apps.
Emotional Impact	Emo_Imp	To describe the participants' emotional reactions related to COVID-19 or the contact tracing device.
**Overarching Codes**		
Successes/Facilitators	Suc_Fac	Things that worked well, positive support.
Challenges/Barriers	Chal_Bar	Things that didn't work well, preventing the project from executing effectively.
Recommendations	Rec	Recommenation for the project or for research team.
Great Quote	Grt_Qot	Anything that might be particularly useful, either to the project or for a future paper/presentation.
