# Supplementary material for: Implementation of a Hardware-Assisted Bluetooth-Based COVID-19 Tracking Device in a High School: Mixed Methods Study
Source: JMIR Form Res. 2023 Apr 7;7:e39765. doi: 10.2196/39765 (PMC10131711; doi:10.2196/39765)
Supplement: Multimedia Appendix 4 [file formative_v7i1e39765_app4.docx]

| Ease of Use | Agree n (%) | Neutral n (%) | Disagree n (%) |
| --- | --- | --- | --- |
| It was easy for me to install the syncing app on the device | 80 (71.4%) | 3 (2.4%) | 29 (26.2%) |
| It was easy for me to learn to use the syncing app | 84 (75.0%) | 9 (8.3%) | 19 (16.7%) |
| The syncing app was easy to use | 68 (60.7) | 5 (4.8%) | 39 (34.5%) |
| The device was convenient to carry with me throughout my school day/while on the school campus | 61 (54.8) | 8 (7.1%) | 43 (38.1%) |
