# Supplementary material for: Implementation of a Hardware-Assisted Bluetooth-Based COVID-19 Tracking Device in a High School: Mixed Methods Study
Source: JMIR Form Res. 2023 Apr 7;7:e39765. doi: 10.2196/39765 (PMC10131711; doi:10.2196/39765)
Supplement: Multimedia Appendix 5 [file formative_v7i1e39765_app5.docx]

| Interface and Satisfaction | Agree n (%) | Neutral n (%) | Disagree n (%) |
| --- | --- | --- | --- |
| I like the interface of the syncing app | 36 (32.2%) | 33 (29.8%) | 43 (38.0%) |
| The information in the syncing app was well organized, so I could easily find the information I needed | 49 (44.0%) | 24 (21.4%) | 39 (34.6%) |
| The syncing app adequately acknowledged and provided information to let me know the progress of my action | 64 (57.1%) | 17 (15.5%) | 31 (27.4%) |
| The amount of time involved in using the syncing app is acceptable | 63 (55.9%) | 9 (8.3%) | 40 (35.8%) |
| I would use the device and syncing app again | 51 (45.4%) | 21 (19.0%) | 40 (35.6%) |
| Overall, I am satisfied with this system | 56 (50%) | 21 (19.0%) | 35 (31.0%) |
