# Supplementary material for: Implementation of a Hardware-Assisted Bluetooth-Based COVID-19 Tracking Device in a High School: Mixed Methods Study
Source: JMIR Form Res. 2023 Apr 7;7:e39765. doi: 10.2196/39765 (PMC10131711; doi:10.2196/39765)
Supplement: Multimedia Appendix 6 [file formative_v7i1e39765_app6.docx]

| Usefulness | Agree n (%) | Neutral n (%) | Disagree n (%) |
| --- | --- | --- | --- |
| The system would be useful for contact tracing | 83 (73.8%) | 12 (10.7%) | 17 (15.5%) |
| Carrying the device with me helped increased my awareness of my social interactions | 42 (37.3%) | 20 (18.1%) | 50 (44.6%) |
| The syncing app has all the functions and capabilities I expected it to have | 52 (46.4%) | 35 (31.0%) | 25 (22.6%) |
