# Supplementary material for: Implementation of a Hardware-Assisted Bluetooth-Based COVID-19 Tracking Device in a High School: Mixed Methods Study
Source: JMIR Form Res. 2023 Apr 7;7:e39765. doi: 10.2196/39765 (PMC10131711; doi:10.2196/39765)
Supplement: Multimedia Appendix 7 [file formative_v7i1e39765_app7.docx]

| Coherence | Agree n (%) | Neutral n (%) | Disagree n (%) |
| --- | --- | --- | --- |
| I understand how data collected with this system would be used for contact tracing | 97 (86.9%) | 7 (6.0%) | 8 (7.1%) |
| I understand how this system currently protects my privacy | 83 (73.9%) | 15 (13.1%) | 15 (13.0%) |
