# Supplementary material for: Implementation of a Hardware-Assisted Bluetooth-Based COVID-19 Tracking Device in a High School: Mixed Methods Study
Source: JMIR Form Res. 2023 Apr 7;7:e39765. doi: 10.2196/39765 (PMC10131711; doi:10.2196/39765)
Supplement: Multimedia Appendix 8 [file formative_v7i1e39765_app8.docx]

| Appropriateness | Agree n (%) | Neutral n (%) | Disagree n (%) |
| --- | --- | --- | --- |
| It is appropriate for your school to use Bluetooth devices to monitor interactions on campus in order to more efficiently perform contact tracing | 90 (80.5%) | 14 (12.2%) | 8 (7.3%) |
| It is appropriate to use location information such as GPS and/or Wi-Fi connection data for contact Tracing | 67 (60.3%) | 18 (15.7%) | 27 (24.1%) |
| I would prefer to use a school-owned contact tracing device as opposed to downloading an app on my personal phone | 54 (48.3%) | 35 (31.0%) | 23 (20.7%) |
| I have concerns about how using this system, or a system like it, could affect my privacy | 52 (46.0%) | 21 (19.0%) | 39 (35.0%) |
