# Supplementary material for: Implementation of a Hardware-Assisted Bluetooth-Based COVID-19 Tracking Device in a High School: Mixed Methods Study
Source: JMIR Form Res. 2023 Apr 7;7:e39765. doi: 10.2196/39765 (PMC10131711; doi:10.2196/39765)
Supplement: Multimedia Appendix 9 [file formative_v7i1e39765_app9.docx]

| **Table S7A.** | Adherence | **Agree n (%)** | **Neutral n (%)** | **Disagree n (%)** |
| --- | --- | --- | --- | --- |
|  | Would carry if larger | 1 (0.9%) | 8 (7.1%) | 103 (93.6%) |
|  | Would carry if smaller | 104 (92.9%) | 7 (6.3%) | 3 (2.7%) |
|  |  |  |  |  |
| **Table S7B.** | **What prevents you from carrying the device?** | **n (%)** |  |  |
|  | Forgot at home/dorm | 19 (57%) |  |  |
|  | Forgot at Desk | 6 (18%) |  |  |
|  | Intentionally left at home/dorm | 2 (6%) |  |  |
|  | Intentionally left at desk or in locker | 1 (3%) |  |  |
|  | Others | 5 (15%) |  |  |
|  |  |  |  |  |
|  |  |  |  |  |
| **Table S7C.** | **How did you carry the device with you throughout the day?** | **n %** |  |  |
|  | Pocket | 9 (28%) |  |  |
|  | Backpack/Purse | 8 (24%) |  |  |
|  | Attached to a lanyard or beit | 6 (18%) |  |  |
|  | Attached to my clothes | 4 (12%) |  |  |
|  | Left on school desk | 2 (6%) |  |  |
|  | Left in the dorm | 2 (6%) |  |  |
|  | Left at home | 1 (3%) |  |  |
|  | Others | 1 (3%) |  |  |
|  |  |  |  |  |
|  |  |  |  |  |
| **Table S7D.** | **For what proportion (%) of your total school days did you have the device either on you or within arms' reach?** | **n %** |  |  |
|  | 100% | 6 (18%) |  |  |
|  | 99-95% | 4 (12%) |  |  |
|  | 94-90% | 3 (10%) |  |  |
|  | 89-85% | 4 (12%) |  |  |
|  | 84-80% | 2 (6%) |  |  |
|  | 79-75% | 2 (6%) |  |  |
|  | 74-70% | 2 (6%) |  |  |
|  | 69-65% | 1 (3%) |  |  |
|  | 64-60% | 0 (0%) |  |  |
|  | 59-55% | 2 (6%) |  |  |
|  | 54-50% | 1 (3%) |  |  |
|  | below 50% | 6 (18%) |  |  |
|  |  |  |  |  |
